# Combined optical line-of-sight and crosslink radiometric navigation for distributed deep-space systems

**DOI:** 10.1038/s41598-023-43339-9

**Published:** 2023-09-27

**Authors:** Stefano Casini, Erdem Turan, Angelo Cervone, Bert Monna, Pieter Visser

**Affiliations:** 1 Space Engineering, Delft University of Technology, Delft, 2629 HS The Netherlands; 2 Phosphoenix, Amsterdam, 1105 AZ The Netherlands

**Keywords:** Aerospace engineering, Imaging and sensing

## Abstract

This manuscript aims to present and evaluate the applicability of combining optical line-of-sight (LoS) navigation with crosslink radiometric navigation for deep-space cruising distributed space systems. To do so, a set of four distributed space systems architectures is presented, and for each of those, the applicability of the combination is evaluated, comparing it to the baseline solutions, which are based on only optical navigation. The comparison is done by studying the performance in a circular heliocentric orbit in seven different time intervals (ranging from 2024 to 2032) and exploiting the observation of all the pairs of planets from Mercury to Saturn. The distance between spacecraft is kept around 200 km. Later, a NEA mission test case is generated in order to explore the applicability to a more realistic case. This analysis shows that the technique can also cope with a variable inter-satellite distance, and the best performance is obtained when the spacecraft get closer to each other.

## Introduction

In recent years, deep-space exploration has seen the rise of new interesting trends. Among them, miniaturized and autonomous satellites occupy a primary role, as they have the capabilities to expand the exploration possibilities for a wide range of deep-space targets, especially asteroids. Moreover, there has been a growing interest in developing distributed space systems (DSS) that can operate in unison to achieve common goals. However, a major challenge in developing such systems is the need for accurate and reliable navigation capabilities, particularly in deep-space cruising scenarios where traditional navigation methods may be limited or ineffective^1^, while in close proximity operations around targets it has been widely investigated. In particular, autonomous navigation for DSS has been analyzed around asteroids, with some techniques capable of estimating unknown asteroid’s parameters together with the satellites’ state^2,3^. This challenge is further complicated when it comes to optimizing the mass and volume distributions, as well as operations planning, for miniaturized satellites, whose resources and capabilities are significantly reduced compared to those of larger spacecraft^4^.

In this context, the integration of multiple navigation techniques has emerged as a promising solution. This paper explores the combination of two distinct navigation methods, namely crosslink radiometric navigation and optical line-of-sight navigation, for a number of DSS consisting of either two or three satellites with varying sensor complements. The former is intrinsically related to the concept of distributed space systems, and it is based on measuring range and/or range-rate information between the satellites. It has been proven to be capable of estimating the satellite state in various range of scenarios, but it lacks of performance when it comes to estimating the state of two or more satellites whose orbits or trajectories are too similar under a dynamics point of view^5^. On the other hand, the latter is a very promising technique capable of estimating the state of a deep-space cruising spacecraft, by only observing the direction of visible celestial bodies with onboard optical instrumentation. Its applicability to deep-space CubeSats has been proved^6^. The technique is also reliable in the exploration of outer Solar System objects, using their moons LoS direction^7^. The drawback is that to achieve low estimation error, the observation of at least two planets is required^8^. This is sometimes complicated for low-resources spacecraft, as it would require either multiple onboard cameras and/or continuous attitude re-orientation to align the planet in the camera Field-of-View (FoV) cone.

The combination of the two for DSS has the advantage of enabling lighter and less complicated architectures, by exploiting the possibilities of exchanging range information among the spacecraft of the network.

The paper presents a detailed description of the proposed navigation strategy, including the design and implementation of the crosslink radiometric and optical line-of-sight navigation methods. The performance of the system is evaluated through simulations and experiments, demonstrating its effectiveness in deep-space cruising scenarios.

Overall, the results presented in this paper suggest that the integration of crosslink radiometric and optical line-of-sight navigation methods can offer significant advantages for distributed space systems, particularly in terms of reducing the onboard equipment required for navigation. This has important implications for the design and operation of future space missions, opening up new possibilities for exploration and scientific research.

The paper is organized as follows: first, the concept of DSS is explained, and the network architectures considered in this study are detailed. Then both navigation techniques are presented, with them the Extended Kalman Filter (EKF) formulation for their combination. Later, a wide range of mission scenarios is considered in order to define the advantages and disadvantages of the combined navigation techniques, by considering circular heliocentric orbits. After, a test mission to a Near-Earth Asteroid (NEA) is generated and the combined navigation technique is tested. Finally, conclusions are reported, focusing on the next steps of the roadmap to enable these missions.

## Distributed space systems architectures

The term DSS encompasses a range of mission concepts that involves the utilization of multiple spacecraft to achieve mission objectives. Constellations, formation flying, and satellite swarms serve as instances of distributed space systems^9^. These systems, such as formations and swarms, present numerous cost benefits and open doors to fresh functional possibilities and improved performance. They also bring forth diverse scientific and engineering challenges. This, in turn, has the potential to result in innovative architectures, disruptive engineering methodologies, and novel technologies. Consequently, these advancements can enable new capabilities, enhance characteristics, and bring about cost reductions. In this paper, the focus is on small satellites, in particular CubeSats, as they have seen in recent years an interest rise, due to their reduced cost compared to standard missions, and due to their design standardization. Nevertheless, the concept of DSS, and therefore the combined navigation, can be also applied to larger spacecraft architectures.

**Figure 1:**
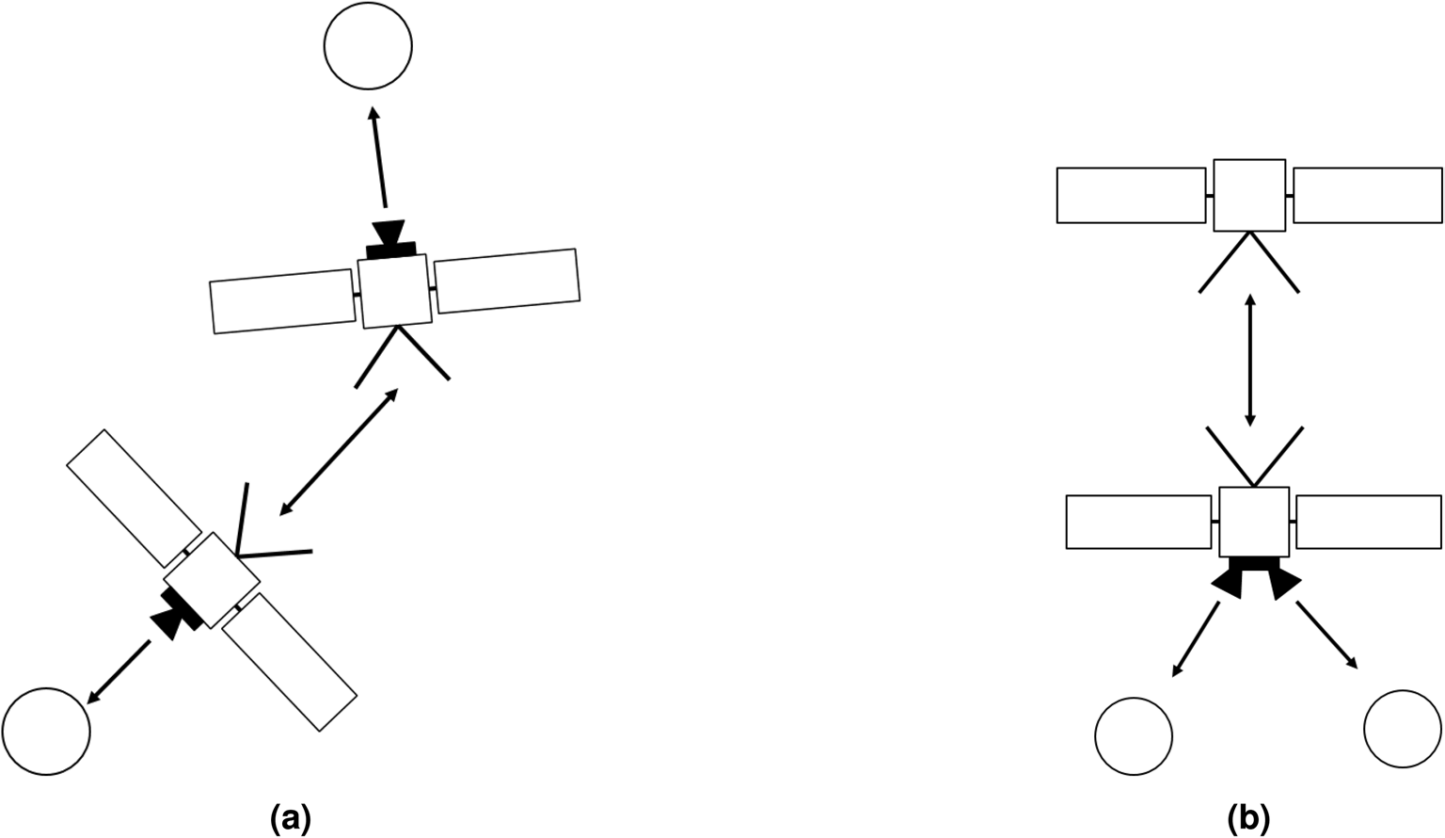
Architecture A with two satellites, each of them equipped with a camera (**a**). Architecture B with two satellites, one equipped with two cameras and one without (**b**). The double side arrows represent the range information.

For these reasons, in this analysis, four different architectures have been considered. The first two architectures are based on a two-satellites network, as shown in Fig. 1. The first architecture involves one camera per satellite and is applicable to mission scenarios that involve two identical CubeSats, such as Marco-A and -B^10^, as well as recent mission proposals like NEOCORE^11^. It is referred to in this paper as architecture A. The second architecture involves two cameras on one spacecraft and none on the other, resembling the mothercraft-daughtercraft concept or a DSS that is entirely split, with the navigation functionality restricted to one segment of the network. This is referred to as architecture B.

**Figure 2:**
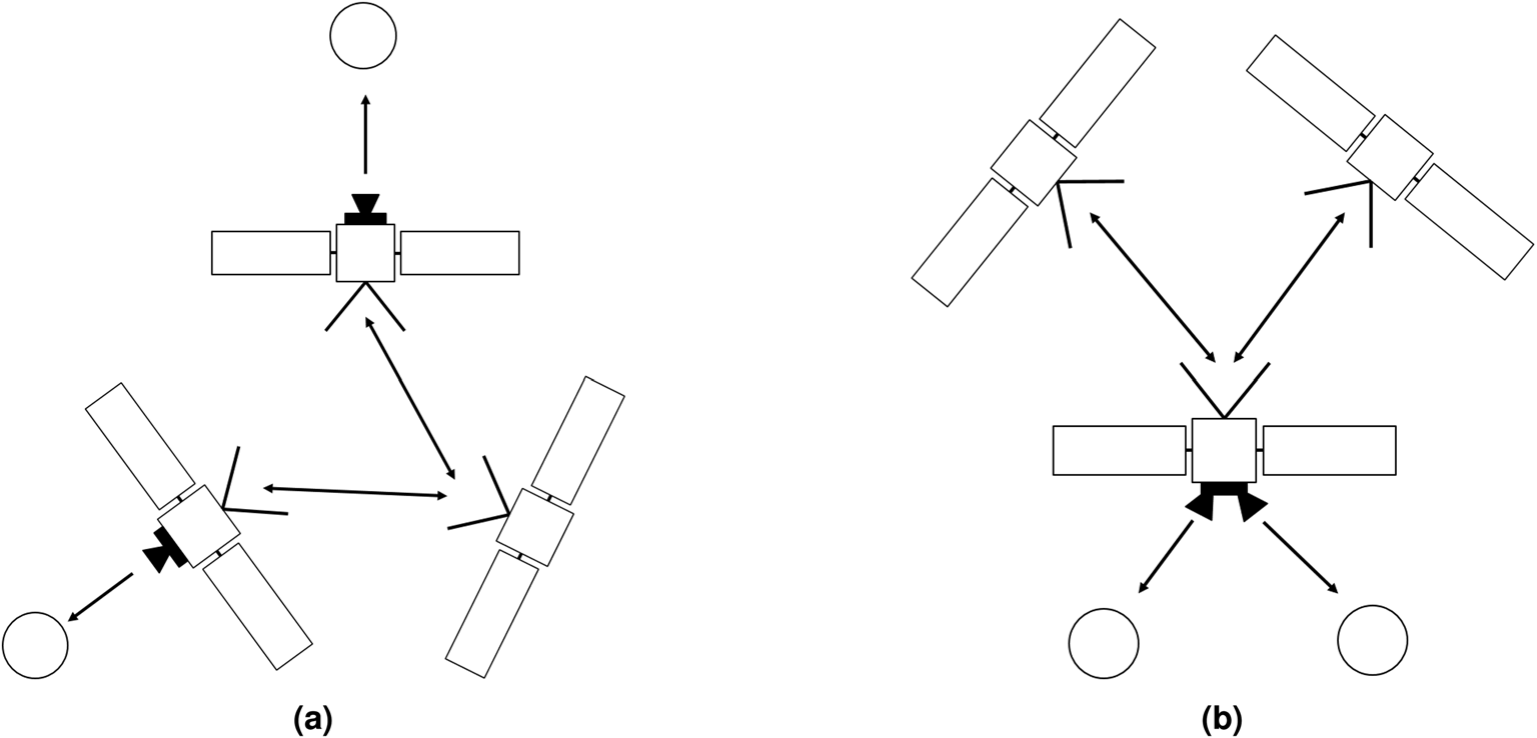
Architecture C with three satellites, two of them equipped with one camera each, and one of them without (**a**). Architecture D with three satellites, one of them equipped with two cameras, and the other two without (**b**). The double side arrows represent the range information.

In the second macro category of architectures, a three satellites network has been considered, as depicted in Fig. 2. The first, namely architecture C, involves a central spacecraft with no cameras onboard, ranging with two other satellites, each of those equipped with a camera. The second, namely architecture D, is characterized by the central spacecraft equipped with two cameras, and two other ranging satellites with no cameras onboard. Both architectures can be associated with DSS where the functions are split among elements, while the second can again refer to the case of a larger spacecraft accompanied by two small satellites. An additional range link can be established between the two side satellites, for both architectures C and D. Its utility is discussed later in the results section, however, it would slightly complicate the operations, since the additional range measurement shall be observed beforehand and then communicated by one of the two side spacecraft to the central one.

## Combined LoS and crosslink radiometric navigation

The goal of this paper is to present the applicability of the combination of two powerful autonomous navigation techniques that can be exploited in deep-space for state estimation. LoS navigation involves the observation of visible celestial objects (mostly planets), whose direction is measured and used to estimate the state of the spacecraft with respect to an inertial frame, where the position of these objects can be easily retrieved. As has been shown by Casini et al.^8^, this technique offers notable better performances (e.g. lower estimation error) if two or multiple planets are observed, while they deteriorate in the case of single planet tracking. This implies that either the satellite should be equipped with two cameras or star-trackers in order to track simultaneously two planets, or that the satellite shall have the capabilities to re-orientate frequently in order to capture in the FoV multiple objects asynchronously. Both these options impact significantly the design of small satellites, as they require either additional hardware, which impacts mass, volume, and power budgets, or the capabilities of the AOCS, which again impact mass and volume budget (as more fuel would be required onboard), as well as complicate the operations. To date, no mission has utilized LoS navigation as the primary technique. However, the CubeSat M-ARGO is anticipated to incorporate an experiment onboard to evaluate its practicality^12^.

On the other hand, crosslink radiometric navigation is a technique based on exchanging range (and eventually range-rate) information between the spacecraft, which are used as measurements for the state estimation within navigation. This technique offers extremely low estimation errors when applied to scenarios where the spacecraft are orbiting around a Lagrangian point or a small Solar System body^5^, especially if the satellites are placed in sufficiently different orbits, characterized by different dynamics, while performance deteriorates when the satellites are orbiting in similar dynamics conditions. This is perhaps the case of cruising towards a deep-space target, as the DSS satellites would travel on sufficiently similar trajectories. This has been demonstrated in Ref.^13^, which shows that the crosslink ranging does not provide full-state estimation in 2-Body Problem (2BP) dynamics. However, in this application, the range measurement is complementary to the planet observations, and it serves conceptually as a link between two or more spacecraft to share their individual planet observation.

For these reasons, this paper proposes to combine the two in order to define a navigation strategy which helps the constrained mission design of miniaturized deep-space DSS, by reducing the mass, volume, power, and operations demand, while still offering low state estimation error.

For this application and analysis, the decision has been made to use a sequential estimation technique instead of a batch approach. The preference for a sequential algorithm stems from its applicability to autonomous deep-space CubeSats. By employing a sequential algorithm, there is a reduced need for onboard computational load, and the state estimation can be continuously updated, allowing for eventual manoeuvres and operations. A formulation based on the EKF^14^ has been developed for addressing the problem of the two-satellites network, but the same rationale can be applied to the three-satellites case. The state to be estimated is the twelve-dimensional Cartesian state expressed in a heliocentric frame (ECLIPJ2000):

1 $$\begin{aligned} \vec {X} = \begin{array}{llllllllllll} [x_1\,\,\,\,\,\,y_1\,\,\,\,\,\,z_1\,\,\,\,\,\,v_{x,1}\,\,\,\,\,\,v_{y,1}\,\,\,\,\,\,v_{z,1}\,\,\,\,\,\,x_2\,\,\,\,\,\,y_2\,\,\,\,\,\,z_2\,\,\,\,\,\,v_{x,2}\,\,\,\,\,\,v_{y,2}\,\,\,\,\,\,v_{z,2} \end{array}]^T. \end{aligned}$$

The equation of the dynamics can be expressed as:

2 $$\begin{aligned} \vec {{\dot{X}}} = f(\vec {X}(t)), \end{aligned}$$

where vector $\vec {{\dot{X}}}$ contains the time-derivatives of the state vector, and it is expressed as a function *f* of the state vector. In this analysis, a 2BP has been considered, in order to simplify the analysis and to focus on the general characteristics:

3 $$\begin{aligned} \vec {\ddot{r}} = - \mu _{s} \cdot \frac{\vec {r}}{r^3}, \end{aligned}$$

where *r* is the 3D position vector, while $\mu _{s}$ is the solar gravitational constant. The measurements fed to the EKF are the directions to one or more planets, expressed in terms of azimuth $\theta$ and elevation $\psi$ with respect to the observer (e.g. the spacecraft), and the range $\rho$ between the satellites:

4 $$\begin{aligned} \vec {Y} = h(\vec {X}(t)) = \begin{bmatrix} \theta _1&\ \psi _1&\ \theta _2&\ \psi _2&\ \rho \end{bmatrix}^T. \end{aligned}$$

The size of the vector $\vec {Y}$ depends on the available measurements, but in this analysis, it has five entries in the two spacecraft case, and six entries in the three spacecraft case, as it is assumed that always two planets are tracked simultaneously. Directions to planets are expressed in the same heliocentric frame as the state, and they can be computed as:

5 $$\begin{aligned} {\hat{r}}_{pk} = \frac{\vec {r}_{k} - \vec {r}}{|\vec {r}_{k} - \vec {r} |} = \begin{bmatrix} x_{los,k} \\ y_{los,k} \\ z_{los,k} \end{bmatrix}, \end{aligned}$$

where $\vec {r}_{k}$ is the heliocentric $k{\text {th}}$-planet position vector. The spacecraft attitude is considered known and it is taken into account to define the LoS measurement error range. Respectively, Azimuth, Elevation, and Range can be computed from the state as follows:

6 $$\begin{aligned}{} & {} \begin{bmatrix} \theta _k \\ \psi _k \end{bmatrix} = \begin{bmatrix} \arctan (\frac{y_{los,k}}{x_{los,k}}) \\ \arcsin {(z_{los,k})} \end{bmatrix}, \end{aligned}$$

7 $$\begin{aligned}{} & {} \rho = \sqrt{(x_1 - x_2)^2 + (y_1 - y_2)^2 + (z_1 - z_2)^2} + \Delta \rho , \end{aligned}$$

where $\Delta \rho$ is range bias, which quantifies instrumental delays, and for this analysis, it has been assumed zero, while for further studies it can be estimated. In practical scenarios, it is necessary to account for light-time delay and velocity aberration corrections. Light-time delay pertains to the fact that it takes a specific duration for light to travel from the observed object to the spacecraft. This duration must be calculated onboard in order to access the ephemerides at the precise time. The computation of light-time delay is also affected by the spacecraft’s onboard timing knowledge. Velocity aberration can be similarly accounted for by calculating the beam shift as a function of the satellite estimated position and velocity^15^. However, in this study including both effects would not add any particular insight, so it has been neglected. Bold characters in the next equations indicate matrices. The state is propagated within the filter by solving the differential Equation (3), while the covariance matrix is propagated as:

8 $$\begin{aligned} \bar{{\varvec{P}}_{k}} = \varvec{\phi }^T(t_k,t_{k-1}) \hat{{\varvec{P}}_{k-1}}\varvec{\phi }(t_k,t_{k-1}) + {\textbf{Q}}, \end{aligned}$$

where $\varvec{\phi }(t_k,t_{k-1})$ is the state transition matrix and $\varvec{Q}$ is the process noise matrix, which is assumed to be diagonal with the position and velocity components values set respectively to $10^{-12}$ km^2^ and $10^{-11}$ km^2^/s^2^. These values have been preliminary tuned and kept small since the dynamics is assumed perfectly modelled within the filter. However, in realistic filter tuning scenarios, attention shall be devoted to their selection in order to account for unmodelled perturbances.

The state transition matrix is computed at each time interval by integrating:

9 $$\begin{aligned} {\dot{\varvec{\phi }}}(t) = \varvec{F} \varvec{\phi }(t), \end{aligned}$$

where $\varvec{F}$ is the Jacobian of the state, and in the 2-body problem formulation can be expressed as:

10 $$\begin{aligned}{} & {} \varvec{J_k} = \begin{bmatrix} 0 &{} 0 &{} 0 &{} 1 &{} 0 &{} 0\\ 0 &{} 0 &{} 0 &{} 0 &{} 1 &{} 0\\ 0 &{} 0 &{} 0 &{} 0 &{} 0 &{} 1\\ - \frac{\mu _s(1 - \frac{3x_k^2}{r_k^2})}{r_k^3} &{} 3 \mu _s \frac{x_k}{r_k^5} &{} 3 \mu _s \frac{x_k z_k}{r_k^5} &{} 0 &{} 0 &{} 0\\ 3 \mu _s \frac{x_k y_k}{r_k^5} &{} - \frac{\mu _s(1 - \frac{3y_k^2}{r_k^2})}{r_k^3} &{} 3 \mu _s \frac{y_k z_k}{r_k^5} &{} 0 &{} 0 &{} 0\\ 3 \mu _s \frac{x_k z_k}{r_k^5} &{} 3 \mu _s \frac{y_k z_k}{r_k^5} &{} - \frac{\mu _s(1 - \frac{3z_k^2}{r_k^2})}{r_k^3} &{} 0 &{} 0 &{} 0 \end{bmatrix}, \end{aligned}$$

11 $$\begin{aligned}{} & {} \varvec{F} = \begin{bmatrix} \varvec{J_1} &{} \varvec{0_{6,6}} \\ \varvec{0_{6,6}} &{} \varvec{J_2}\\ \end{bmatrix}. \end{aligned}$$

The state vector and covariance matrix update can be then expressed as:

12 $$\begin{aligned}{} & {} \vec {{\hat{X}}}_{k} = \vec {{\bar{X}}}_{k} + \varvec{K_{k}}(\vec {Y}_{k}- \vec {{\bar{Y}}}_{k}), \end{aligned}$$

13 $$\begin{aligned}{} & {} \varvec{{\hat{P}}_{k}} = \bigg ({\varvec{I}}- \varvec{K_{k}}\varvec{H_{k}}\bigg )\varvec{{\bar{P}}_{k}}, \end{aligned}$$

where $\vec {{\bar{X}}}_k$ is the propagated state vector, $\vec {{\hat{X}}}_k$ is the updated state vector, $\vec {Y}_k$ is the measurements vector, $\vec {{\bar{Y}}}_k$ is the computed measurements vector, $\varvec{{\bar{P}}_{k}}$ is the propagated covariance matrix, $\varvec{{\hat{P}}_{k}}$ is the updated covariance matrix. Respectively, the observation matrix $\varvec{H_k}$ and the Kalman gain matrix $\varvec{K_k}$ can be expressed as:

14 $$\begin{aligned}{} & {} \varvec{C_{k,i}} = \begin{bmatrix} \frac{y_{los,i}}{r_{los,i}^2} &{} - \frac{x_{los,i}}{r_{los,i}^2} &{} 0 &{} 0 &{} 0 &{} 0\\ \frac{x_{los,i}z_{los,i}}{r_{los,i}^3\sqrt{1- \frac{z_{los,i}^2}{r_{los,i}^2}}} &{} \frac{y_{los,i}z_{los,i}}{r_{los,i}^3\sqrt{1- \frac{z_{los,i}^2}{r_{los,i}^2}}} &{} \frac{z_{los,i}^2-r_{los,i}^2}{r_{los,i}^3\sqrt{1- \frac{z_{los,i}^2}{r_{los,i}^2}}} &{}\ 0 &{}\ 0 &{}\ 0\\ \end{bmatrix}, \end{aligned}$$

15 $$\begin{aligned}{} & {} \Gamma = \begin{bmatrix} \frac{x_1 - x_2}{\rho } &{} \frac{y_1 - y_2}{\rho } &{} \frac{z_1 - z_2}{\rho } &{} 0 &{} 0 &{} 0\\ \end{bmatrix}, \end{aligned}$$

16 $$\begin{aligned}{} & {} \varvec{H_k} = \begin{bmatrix} \varvec{C_{k,1}} &{} \varvec{0_{2,6}} \\ \gamma \cdot \varvec{C_{k,1}} &{} \delta \cdot \varvec{C_{k,1}} \\ \Gamma &{} - \Gamma \\ \end{bmatrix},\end{aligned}$$

17 $$\begin{aligned}{} & {} \varvec{K_{k}} = \varvec{{\bar{P}}_{k}}\varvec{H^T_{k}}(\varvec{H_{k}}\varvec{{\bar{P}}_{k}}\varvec{H^T_{k}} + \varvec{R_{k}})^{-1}, \end{aligned}$$

where $\gamma$ is set to 0 when architecture A is analyzed and to 1 for B, while $\delta$ is set conversely, and $\varvec{R_k}$ is the observation covariance matrix, assumed constant in this analysis:

18 $$\begin{aligned} \varvec{R_k} = \begin{bmatrix} \sigma _{los1}^2 &{} 0 &{} 0 &{} 0 &{} 0\\ 0 &{} \sigma _{los1}^2 &{} 0 &{} 0&{} 0\\ 0 &{} 0 &{} \sigma _{los2}^2 &{} 0 &{} 0\\ 0 &{} 0 &{} 0 &{} \sigma _{los2}^2 &{} 0\\ 0 &{} 0 &{} 0 &{} 0 &{} \sigma _{\rho }^2 \end{bmatrix}, \end{aligned}$$

where the diagonal values correspond to the standard deviations of the measurements.

These equations apply to a two-spacecraft architecture, however, the equations for the three spacecraft cases follow the same rationale and can be easily derived, but they are not shown in this paper.

LoS azimuth and elevation standard deviations mainly depend upon two main players: the attitude estimation accuracy that is used to convert the LoS direction from body-fixed to an inertial frame, and the planet centroiding accuracy, which is influenced by the observation scenario (e.g. distance and illumination condition from the planet), hardware characteristics, and centroiding algorithm. In this analysis, $\sigma _{los} = 5 arcsec$ is assumed as attitude estimation accuracy using CubeSat star-trackers ranges between a few to tens of arcsecs depending on both hardware characteristics and algorithms^16^.

Several methods exist for inter-satellite radiometric ranging, including conventional PN/Tone, telemetry, and frame ranging. In this study, telemetry-ranging has been adopted as the inter-satellite ranging method due to its high telemetry data transmission rate between satellites. Telemetry ranging does not rely on a separate downlink ranging signal and is dependent on the timestamping and identification of synchronized information frames. According to CCSDS 401.0-B-32^17^, telemetry ranging can achieve range accuracy of 1 m ($1\sigma$) when the coded symbol rate is 200ksymbol/s or greater under the specific ground station and spacecraft conditions. A high telemetry data rate can be advantageous for this ranging method due to the shorter symbol duration, $T_{sd}$. With inter-satellite distances around 200 km considered in this study, hundreds of kbps of data rates can be easily achieved. Moreover, using a telemetry window for navigation purposes enables constant tracking between satellites without requiring a separate time window for tracking. Its performance could be defined as follows^18^:

19 $$\begin{aligned} \sigma _{\rho _{SST}} = \sigma _{\rho _{TM}} = \left( 1- \frac{2v}{c} \right) \left( \frac{4 \, c \, T_{sd}^{2}}{\pi \, T_{l} \, E_{S}/N_{0}} + \frac{c}{8 f_{rc}}\sqrt{\frac{B_{L}}{(P_{RC}/N_{0})}} \right) , \end{aligned}$$

where the telemetry symbol duration is denoted as $T_{sd}$, *c* represents the speed of light, *v* is the relative velocity between satellites, $T_{l}$ is the correlator integration time, $E_{S}/N_{0}$ denotes the symbol-to-noise ratio, $f_{rc}$ represents the frequency of the ranging clock component, $P_{RC}/N_0$ is the signal power to noise spectral density ratio, and $B_{L}$ denotes the one-sided loop noise bandwidth. It is assumed that the onboard time-tagging, related to the master clock frequency, is sufficiently precise and will not affect the overall system performance.

**Figure 3:**
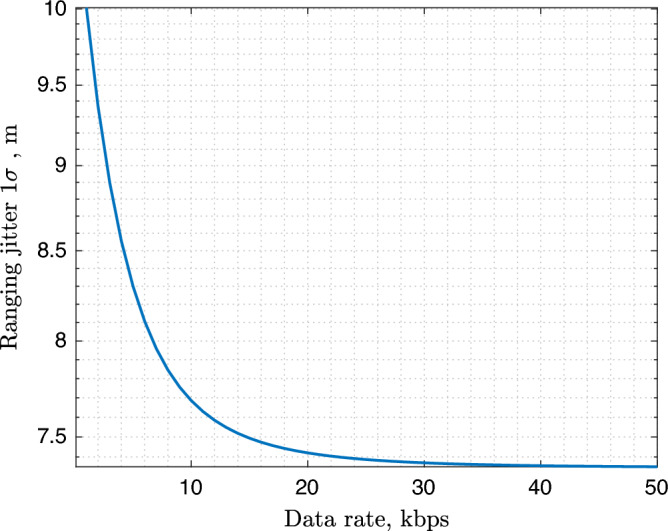
Expected Inter-satellite range measurement error as a function of the inter-satellite data-rate.

For this analysis, the radiometric parameters are reported in Table 1 accordingly to CubeSat characteristics. It is assumed that the link established between the DSS does not rely on X-Band, which is usually devoted to Earth communication. The given configuration provides around ~ 7–8 m $(1\sigma )$ inter-satellite ranging error so that the EKF is set with $\sigma _{\rho } = 10$m, in order to take into account eventual unmodelled disturbances and to be more conservative with the results. Figure 3 shows the expected range error as a function of the inter-satellite data-rate.

**Table 1:** Crosslink characteristics.

	Downlink	Uplink
Frequency	2200 MHz	2100 MHz
TX power	3 dBW	3 dBW
TX path losses	1 dB	1 dB
TX antenna gain	6.5 dBi	6.5 dBi
Data rate (max dist.)	10 kbps	10 kbps
Required Eb/N0	2.5 dB	2.5 dB
Link margin	3 dB	3 dB
Range clock frequency $f_{rc}$	N/A	1 MHz
Integration time $T_l$	1 s	1 s
$P_{RC}/N_0$	N/A	20 dBHz

### Performance of the two spacecraft DSS architecture

As previously discussed, the two satellite architectures are mainly associated with two mission scenarios: a pair of deep-space cruising CubeSats (or more generally SmallSats), and the mothercraft–daughtercraft configuration. Since the objective of this paper is to compare the performance of combined navigation with respect to standalone optical navigation, the results are presented as a comparison between singular planet observation, double planet observation, and combined navigation. Singular and double planets observation cases are referred to in the paper as baseline solutions. However, due to the constantly changing observation geometry, it is not easy to define the steady-state behaviour for LoS navigation, and this generates oscillation in the estimation error. Therefore, presenting the results on a wide-scale comparison is not possible. Thus, this paper chooses to present the results as the difference between the mean 3D position error between the techniques. Keeping this in mind, the mean error is calculated after an ’assessment period’ of 200 days. This number is a good trade-off for displaying results and is chosen based on the convergence time analysis reported in Ref.^8^.

For the purpose of this analysis, a circular heliocentric reference orbit with a radius of 1 AU has been considered. This choice is based on the definition of NEAs, which are characterized by having a perihelion distance of up to 1.3 AU and an aphelion distance of at least 0.983 AU^19^, so missions towards these bodies are expected to happen in this portion of the Solar System. The inter-satellite range is fixed at 200 km throughout all simulations. Results are averaged Root-Mean-Square Error (RMSE) of a Monte Carlo Simulation with 50 trials and then presented as percentage change with respect to a baseline solution. Initial position and velocity dispersions are set respectively to $10^3$ km and 0.001 km/s.

**Figure 4:**
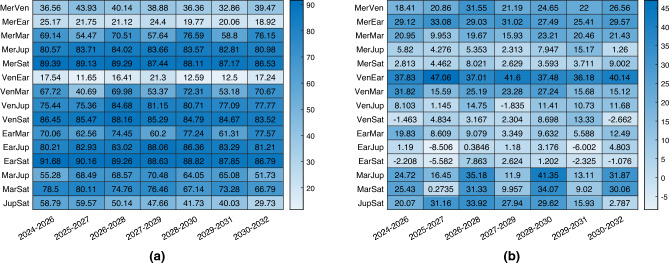
Percentage change in 3D position estimation error between combined navigation architecture A and optical navigation with only one planet. In (**a**) the one planet is the second, while in (**b**) is the first.

The results for architecture A are presented in Fig. 4, and in most cases, the combined navigation approach shows higher accuracy. The table shows the percentage improvement or worsening between the singular planet baseline solution and the combined navigation, so the larger the value, the larger the performance improvement with the combined navigation technique. However, whether the improved accuracy is worthwhile or not depends on the specific mission characteristics and spacecraft availability. It is noteworthy to remark on how the improvement in the estimation error is more dominant when outer planets are observed, and this is of course connected to the results presented in Ref.^8^, where it is shown how the observation of farther planets usually leads to worse performance. So, when only outer planets are available to observe, the combined navigation strategy increases notably the performance. On the other hand, when observing closer bodies, on average the combined navigation still offers improved performance in the order of some hundreds of kilometers, which for asteroid missions can be fundamental. It has to be remarked that there are few cases characterized by a low worsening of performance. This happens with outer planets Jupiter and Saturn, which are sometimes associated with performance lowering even in the baseline solutions when their position is not convenient for optical navigation.

**Figure 5:**
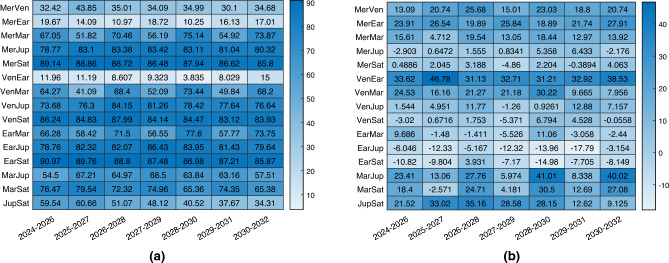
Percentage change in 3D position estimation error between combined navigation architecture B and optical navigation with only one planet. In (**a**) the one planet is the second, while in (**b**) is the first.

The results for architecture B are shown in Fig. 5. The spacecraft for which the results are displayed is the one not observing any object. Again, the results are particularly improved in most of the scenarios, or at least similar. Nevertheless, the crucial outcome is the ability to estimate the second spacecraft’s state even without direct observation of any planet.

**Figure 6:**
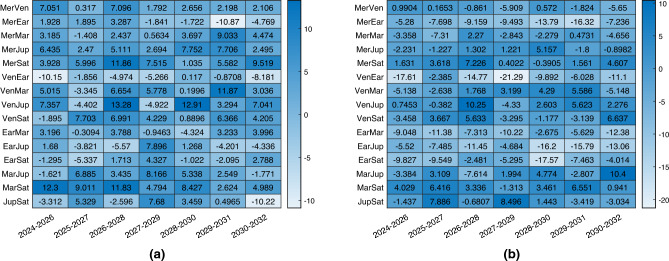
Percentage change in 3D position estimation error between combined navigation and optical navigation with only two planets. Architecture A in (**a**), architecture B in (**b**).

The results of the comparison with the observation of two planets are shown in Fig. 6. On the left, the comparison is for architecture A, which is characterized by a very similar performance as hoped. Then each scenario is characterized by a different behaviour, but the difference between the two is usually contained within 100 km. On the right, the comparison is for architecture B, whose performance is usually slightly worst, even if in some cases they are better. However, in this case, it is impressive to see how the spacecraft not observing any object, simply by ranging with the other companion, is able to estimate almost with the same accuracy its position in deep-space. This aligns with the objective of this paper, which isn’t to demonstrate that the combined navigation technique outperforms sole optical navigation. Instead, it highlights that diminishing the necessary count of onboard sensors still permits achieving similar performance.

### Performance of the three spacecraft DSS architecture

As has been previously mentioned, a DSS composed of three spacecraft can be associated mainly with two applications: a larger spacecraft with two small satellites as companions, and a fleet of small satellites. The results of architecture D are very similar to those of architecture B, both if the navigation filter is based on integrating all three members in the estimation, and if the estimation technique is based on separating the analysis into two simulations similar again to architecture B. For this reason, this subsection is aimed to show the performance of architecture C. In this case, it is interesting to explore the performance of the central spacecraft, which is the one not directly observing any visible planet but is only sharing range information with its two companions, both of them capable of observing two different visible bodies. Architecture C+ is defined as architecture C with the addition of a third range link between the two lateral satellites, whose distance is fixed at 400 km.

**Figure 7:**
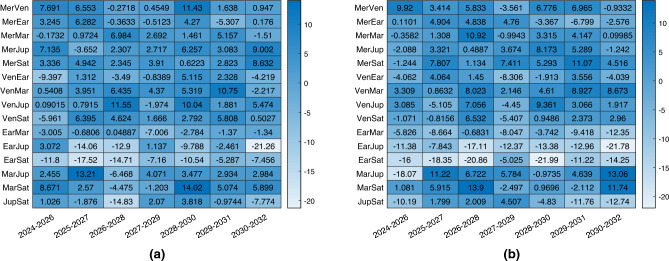
Percentage change in 3D position estimation error between combined navigation and optical navigation with only two planets. Architecture C in (**a**), architecture C with additional range in (**b**).

Results are reported in Fig. 7, where it is shown that architecture C (with and without the additional third range link) is capable of offering comparable performance to the baseline navigation strategy observing two planets. In the majority of the cases, the error is slightly higher than the baseline, however in this case the spacecraft under analysis does not observe directly any planet, simplifying notably the budgets. There is no evident and systematic improvement or deterioration of the performance with the addition of the third link, and this really depends on the scenarios.

It is important to report that this architecture is on the limit concerning observability, with the estimation of an 18-components state with only six or seven measurements. This has an impact when the initial error in position and velocity is larger than the one assumed in this analysis, and it leads to some scenarios in which convergence is not always guaranteed. This problem is however mitigated if the third range is added in the loop, but in general, is less stable than the two-satellites network.

### Mission to NEA example

**Figure 8:**
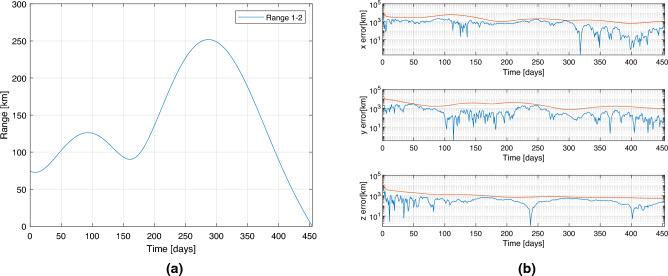
Range profile in (**a**). 3-components position estimation for the single satellite equipped with two cameras, observing Mars and Venus (**b**).

This section is aimed to show the results of a test case based on the exploration of NEA. The target asteroid is 2008 UA202, and to design the trajectory, Lambert’s problem has been solved, considering a release delay of 45 min which generates the range behaviour reported in Fig. 8a. The time-of-flight for this mission is 455 days, identified with a semi-grid search in order to retrieve the propulsion cheaper option. Figure 8b shows the three position components estimation in the case of a standalone satellite capable of tracking two planets. This is the result of one simulation, while later in the section the results are presented as averaged Monte Carlo output. To have a properly tuned filter, it is needed that the error is always contained within the $3\sigma$ boundaries indicated by the red lines.

**Figure 9:**
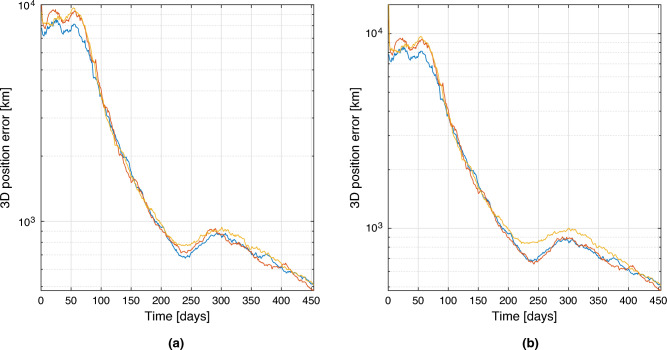
3D Position estimation error for spacecraft 1 (**a**) and spacecraft 2 (**b**). In blue baseline solution, in red architecture A, in yellow architecture B.

**Figure 10:**
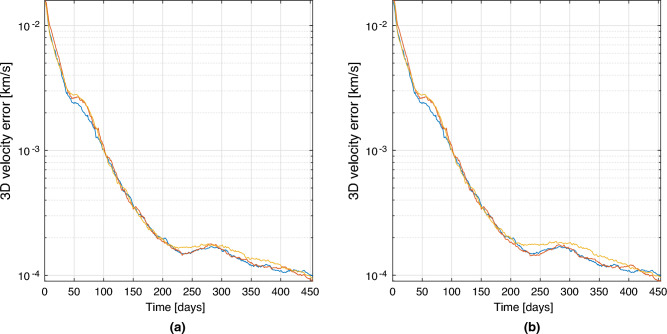
3D Velocity estimation error for spacecraft 1 (**a**) and spacecraft 2 (**b**). In blue baseline solution, in red architecture A, in yellow architecture B.

The observation of Mars and Venus is selected for this example, as it usually represents the optimal observation strategy for a NEA mission. Results of the comparison are presented in the form of average 3D position and 3D velocity error respectively in Figs. 9 and 10, where the errors are compared between the baseline two bodies observation (in blue), the architecture A (in red), and the architecture B (in yellow). The errors are computed as the averaged RMSE of a Monte Carlo simulation with 100 trials. Position and velocity errors present similar trends. As can be noticed in the left plots, after the convergence, the three strategies present almost overlapping behaviours as predicted in the sections before. The results are slightly different for the plots on the right, for which the error in the architecture B is slightly higher, while at the end (when the range between the satellites becomes minimum) it overlaps again. This is expectable as spacecraft 2 in the architecture B is not able to directly observe any objects, and its state estimation relies exclusively on the range information with the other spacecraft. So, even without an inertial information on the second satellite, the inertial position and velocity components can be estimated pretty accurately. These results are just an example of the power of this technique, which shows that even in variable range scenarios, the technique is capable of reconstructing the state of the cruising spacecraft comparably to what it would have been able to estimate alone, if equipped with multiple cameras to observe a pair of planets.

## Conclusions

The paper presents an assessment of combining line-of-sight optical navigation and crosslink radiometric navigation in deep-space cruising scenarios for DSS. The evaluation of this combined technique is deemed necessary as it enables a wide range of mission architectures, and allows the elements of the DSS to be lighter and more compact. In fact, inter-satellite link is assumed to be always established in these applications, and it is used to exchange range information, which compensates for the need to observe multiple objects with the same satellite. In this way, the architecture of the single satellite can be streamlined, allowing more compact architectures in terms of volume, mass, and power consumption. Moreover, it helps also reduce the required AOCS capabilities, as each satellite does not need to constantly re-orientate in order to aline in its FoV with a different object.

First in a general framework, considering a circular heliocentric orbit, and by fixing the range at 200 km, the performance is assessed, showing how this combined technique offers significantly better performance than the ones obtained by observing one object per each satellite, and comparable depending on the scenario with the ones achieved with the observation of the same pair of planets with the same satellite. After this, a test mission to a NEA is generated in order to simulate a variable range, and also in this case the results show that the combined navigation offers very promising performance.

The results show that simply by exchanging the range information between the satellites of the network, even a blind satellite can estimate with comparable accuracy its position and velocity in deep-space, and the same applies to the case of satellites observing only one planet each. In all the architectures, the combined navigation technique shows promising performance, that allows to reduce the mass, volume, power, and operations demand. However, while for the two-satellites network, the technique presents very stable performance, in the three-satellite network, the larger number of states to be estimated with few measurements, generate some instability when the initial position and velocity errors are too large (e.g. larger than $10^3$ km). This effect is slightly mitigated if architecture C+ is considered.

Moreover, the results presented in this paper are obtained in a wide range of scenarios, where general trends have been highlighted. However, navigation geometry is always an important factor leading the performance. In fact, there is a restricted number of scenarios where the combined technique offers slightly lower performance than the singular planet observation baseline. This testifies again that each mission profile has its optimal navigation strategy, which in the large majority of the cases corresponds to the combined technique, but the baseline strategy shall always be investigated.

It is crucial to emphasize that the analyses presented in this paper rely on simplified dynamics and methods, aiming to demonstrate the feasibility of the concept. Subsequently, it becomes necessary to examine how perturbations and deviations from ideal conditions affect the models. Specifically, there is a need to outline the strategy for acquiring measurements, both range and the planet’s LoS direction, as well as for facilitating information exchange among the spacecraft within the system. Additionally, it is imperative to evaluate the consequences of potential delays, as performing entirely simultaneous operations is unfeasible.

To conclude, the analysis presented in this paper presents a first proof of concept in combining navigation techniques for DSS deep-space mission, and in the near future, it would be wise to expand the set of analyses to conclusively prove the applicability and capabilities of this approach that can broaden significantly the opportunities for deep-space exploration.

## Data Availability

Data underlying the results presented in this paper may be obtained from the authors accordingly to TU Delft Data Management Plan.
